# Efficiency and safety of single anastomosis sleeve ileal (SASI) bypass in the treatment of obesity and associated comorbidities: a systematic review and meta-analysis

**DOI:** 10.1007/s00423-024-03413-w

**Published:** 2024-07-18

**Authors:** Carolina Rodrigues Oliveira, Hugo Santos-Sousa, Maria Pinho Costa, Filipe Amorim-Cruz, Raquel Bouça-Machado, Jorge Nogueiro, Fernando Resende, André Costa-Pinho, John Preto, Eduardo Lima-da-Costa, Silvestre Carneiro, Bernardo Sousa-Pinto

**Affiliations:** 1 Faculty of Medicine, University of Porto - Alameda Prof.Hernâni Monteiro, Porto, 4200-319 Portugal; 2 Obesity Integrated Responsibility Unit (CRI-O), São João University Medical Center, Alameda Prof. Hernâni Monteiro, Porto, 4200- 319 Portugal; 3 MEDCIDS - Department of Community Medicine, Information and Health Decision Sciences, Faculty of Medicine, Rua Dr. Plácido da Costa, Porto, 4200-450 Portugal; 4 CINTESIS - Center for Health Technologies and Services Research, University of Porto, Rua Dr. Plácido da Costa, Porto, 4200-450 Portugal; 5 São João University Medical Center, Alameda Prof. Hernâni Monteiro, Porto, 4200-319 Portugal; 6 Instituto de Medicina Molecular João Lobo Antunes, Edifício Egas Moniz, Avenida Professor Egas Moniz, Lisbon, Lisboa, 1649-028 Portugal; 7 Surgery Department, São João University Medical Center, Alameda Prof. Hernâni Monteiro, Porto, 4200-319 Portugal

**Keywords:** Obesity, Bariatric surgery, SASI bypass, Single anastomosis sleeve ileal bypass, Systematic review, Meta-analysis

## Abstract

**Introduction:**

The Single Anastomosis Sleeve Ileal (SASI) bypass is a new bariatric surgery corresponding to an adaptation of the Santoro approach, consisting of a sleeve gastrectomy (SG) followed by loop gastroileostomy. Therefore, we aimed to systematically assess all the current literature on SASI bypass in terms of safety, weight loss, improvement in associated comorbidities, and complications.

**Methods:**

Following the Preferred Reporting Items for Systematic Reviews and Meta- Analyses (PRISMA) recommendations, we conducted a systematic review and meta-analysis by searching three databases (PubMed, Scopus, and Web of Science). We performed a meta-analysis of risk ratios and mean differences to compare surgical approaches for excessive weight loss, improvement/remission in type 2 diabetes mellitus (T2DM), hypertension (HT), dyslipidemia (DL), obstructive sleep apnea (OSA), and complications. Heterogeneity was assessed using the I^2^ statistic.

**Results:**

Eighteen studies were included in the qualitative analysis and four in the quantitative analysis, comparing SASI bypass with SG and One-Anastomosis Gastric Bypass (OAGB). A comparison between Roux-en-Y Gastric Bypass (RYGB) and SASI bypass could not be performed. Compared to SG, the SASI bypass was associated with improved weight loss (MD = 11.32; 95% confidence interval (95%CI) [7.89;14.76]; *p* < 0.0001), and improvement or remission in T2DM (RR = 1.35; 95%CI [1.07;1.69]; *p* = 0.011), DL (RR = 1.41; 95%CI [1.00;1.99]; *p* = 0.048) and OSA (RR = 1.50; 95%CI [1.01;2.22]; *p* = 0.042). No statistically significant differences in any of the assessed outcomes were observed when compared with OAGB. When compared to both SG and OAGB, the complication rate of SASI was similar.

**Conclusion:**

Although studies with longer follow-up periods are needed, this systematic review and meta-analysis showed that SASI bypass has a significant effect on weight loss and metabolic variables. Variations in outcomes between studies reinforce the need for standardization.

**Supplementary Information:**

The online version contains supplementary material available at 10.1007/s00423-024-03413-w.

## Introduction

With the growing prevalence of obesity, it has become a major public health concern [1]. Conservative treatment typically fails to provide satisfactory results. However, bariatric surgery has proven to be the most effective treatment [2].

The most common procedure is the Sleeve Gastrectomy (SG) followed by Roux-en-Y Gastric Bypass (RYGB) [3].

In 2014, Mui et al. described a new technique that was adapted from the Santoro approach, which consisted of SG followed by loop gastroileostomy 250 cm proximal of the ileocecal valve [4]. In 2016, Mahdy et al. presented the first case series of 50 patients with 1-year follow-up and named it the Single Anastomosis Sleeve Ileal (SASI) bypass [5]. By changing the Roux-en-Y anastomosis of Santoro to a simple loop, the SASI bypass appears to be a simpler and safer technique without losing benefits in the treatment of obesity and its comorbidities.

Several studies evaluated the efficacy and safety of this technique. The common limb length varied between 200 and 350 cm. Due to this variation, some authors referred to this technique as “Laparoscopic sleeve gastrectomy with loop bipartition” [6, 7], “Laparoscopic sleeve gastrectomy with loop gastroileal bypass” [8] and “Laparoscopic sleeve gastrectomy with transit loop bipartition” [9].

Therefore, we aimed to systematically assess the current literature on SASI bypass in terms of safety, weight loss, improvement in associated comorbidities, and complications. Additionally, we aimed to establish a comparison with other bariatric surgeries. To the best of our knowledge, this is the first meta-analysis comparing this technique with other bariatric procedures. This study will contribute to a better understanding of this technique and will allow standardization for improved outcomes in the future.

## Methods

This systematic review and meta-analysis was conducted by the Preferred Reporting Items for Systematic Reviews and Meta-analyses (PRISMA) statement guidelines [10].

### Eligibility of primary studies

We included studies assessing adult patients with obesity who underwent SASI bypass. We included both single-arm studies and experimental or observational studies comparing SASI bypass with other bariatric surgeries.

The following outcomes were considered: weight loss and change in body mass index (BMI), improvement in comorbidities such as type 2 diabetes mellitus (T2DM), hypertension (HT), dyslipidemia (DL), gastroesophageal reflux disease (GERD), obstructive sleep apnea (OSA), and complications.

We excluded animal studies, unrelated articles, editorials, correspondence, video reports, reviews, and meta-analyses. In addition, we excluded studies assessing less than 10 participants.

### Search strategy

In October 2022, a systematic review of the available literature was conducted using three search databases - PubMed, Scopus, and Web of Science. The search queries are listed in Supplementary Table 1. In addition, reference lists of the primary articles were hand-searched.

We considered only studies published after 2013, as this technique was first described in 2014 by Mui et al. [4].

### Study selection

After removing the duplicates, study selection was conducted independently by two authors (C.O. and M.O.) in two separate phases. Firstly, the articles were searched by title and abstract. Subsequently, articles not excluded were selected based on their full-text reading. Any disagreements were discussed between the reviewers and a third author (H.S.S.).

### Data collection process

Data were independently extracted by two authors (C.O. and M.O.) into a predesigned data extraction form that was developed according to the Cochrane Handbook [11] to extract relevant information such as author name, publication year, country and journal of the publication, study design, duration of follow-up, sample size, PICO, patient inclusion and exclusion criteria, participants characteristics (mean age, percentage of each gender, mean BMI, number of patients with T2DM, HT, DL, GERD and OSA), interventions, specifics measures of the SASI bypass, outcomes measures (weight loss and improvement in comorbidities), and every kind of complications after surgery.

### Quality assessment

The quality of the studies was independently assessed by two reviewers (M.C. and C.O.) using the National Institutes of Health quality assessment criteria for Observational studies [12], and the Cochrane Collaboration Risk of Bias Tool for randomized control trials (RCT) [13].

The first tool is 14 questions of yes or no, resulting in a final rating (Good, Fair, or Poor). Because exposure is a surgical procedure that can’t be quantified or measured more than once, questions assessing these parameters were considered not applicable.

The second tool classifies each study as having a high, low, or unclear risk bias, based on selection, performance, detection, elimination, reporting, and other bias factors.

### Quantitative synthesis of results

We performed a random-effects meta-analysis (using the restricted maximum likelihood approach) of mean differences (MD) for the continuous outcome (percentage of excess weight loss, %EWL) and relative risks (RR) for dichotomous variables (improvement or remission of T2DM, HT, DL, OSA, and complications).

Heterogeneity was evaluated using Cochran’s Q statistic p-value and the *I*^*2*^ statistic. Heterogeneity was considered high if *I*^*2*^ > 50% or Cochran’s Q test p-value < 0.10. Sources of heterogeneity were studied using leave-one-out sensitivity analysis.

A p-value lower than 0.05 was considered statistically significant. The meta package of software R was used to analyse the data for the meta-analysis.

%EWL is a measured that is calculated as [(preoperative weight–weight on follow-up)/ (preoperative weight - ideal weight)] x 100, with an ideal weight corresponding to an BMI of 25 kg/m^2^.

## Results

### Study selection

**Fig. 1:**
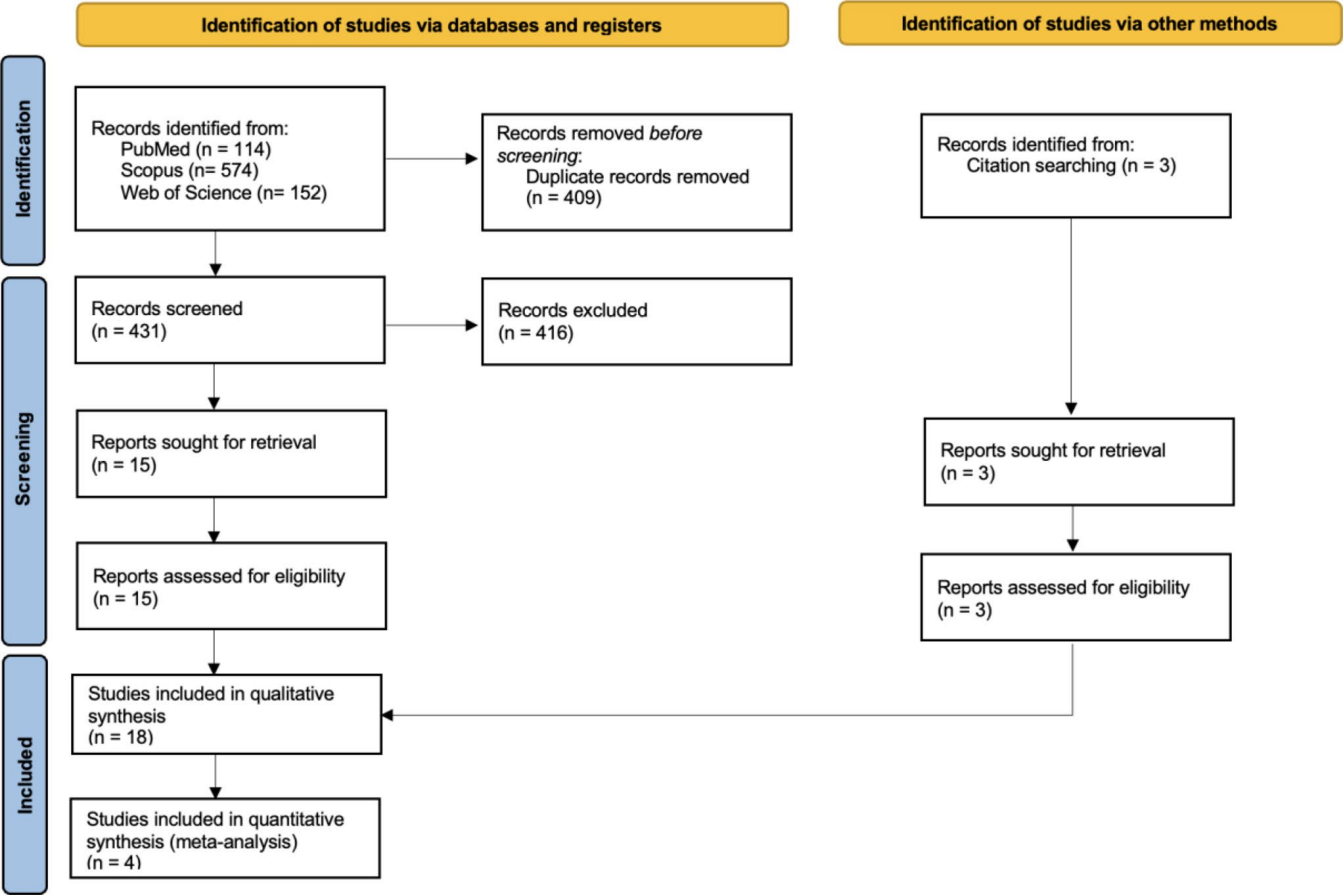
Flow diagram of study selection

After the search in the databases, a total of 840 studies were found, of which 409 were duplicates. After the screening phase, 15 articles were fully read, and all were included. Hand searching resulted in 3 additional articles. A total of 18 articles were included in the qualitative synthesis and 4 in the quantitative synthesis. The study selection process is summarized in Fig. 1 [10].

### Study characteristics

Table 1 shows the characteristics of studies included in the systematic review. Of the 18 articles included 13 were retrospective observational studies [5, 7, 9, 14–23], 4 prospective observational studies [8, 24–26], and 1 RCT [6]. Five studies made a comparison with SG (3 retrospective, 1 prospective, 1 RCT) and 2 retrospective studies made a comparison with OAGB (Table 1).

**Table 1:** Studies included in the systematic review and characteristics of patients who underwent SASI bypass

Author, year	Country	Study Design	Sample size, nº	Female (%)	Preoperative BMI (kg/m^2^), mean ± SD		Follow-up (months)	Common limb, cm	Compares with (nº)
Mahdy et al. [5]	Egypt	Retrospective cohort	50	66	48.7 ± 7.6	12		250	-
Salama et al. [24]	Egypt	Prospective cohort	45	75.5	43.2	12		300	-
Vennapusa et al. [8]	India	Prospective cohort	113	41.6	43.48 ± 7.57	12		250, 300 or 350*	-
Arslan et al. [7]	Turkey	Retrospective cohort	15	46.7	35.05 ± 4.15	3		300	-
Abouzeid et al. [25]	Egypt	Prospective cohort	20	80	45.80 ± 7.60	12		250	-
Khalil et al. [6]	Egypt	Randomized control trial	26	73.1	45.5 ± 7.1	12		250	SG (25)
Mahdy et al. [18]	Multicenter	Retrospective cohort	551	70.8	43.2 ± 12.5	12		250	-
Mohamed et al. [9]	Bahrain	Retrospective cohort	34	-	58.3	12		250	SG (104) and OAGB (39)
Kermansaravi et al. [22]	Iran	Retrospective cohort	24	79.2	44.2 ± 4.3	12		250	-
Madyan et al. [26]	Egypt	Prospective cohort	20	85	53.7 ± 5.9	12		300	SG
Khalaf et al. [20]	Egypt	Retrospective cohort	322	61.5	50.1 ± 7.7	24		250 or 300 **	-
Romero et al. [23]	Mexico	Retrospective cohort	83	63.8	40.9 ± 7.1	12		300	-
Mahdy et al. [17]	UAE	Retrospective cohort	46	50	44.4 ± 9.8	12		300	RYGB (46)
Mahdy et al. [19]	UAE	Retrospective cohort	74	70.3	42.1 ± 14.5	12		300	SG (99) and OAGB (91)
Emile et al. [16]	Multicenter	Retrospective cohort	58	77.6	48.9 ± 16.9	12		250	SG (58)
Tarnowski et al. [21]	Poland	Retrospective cohort	19	94.7	40.3 ± 3.74	12		300	-
Hosseini et al. [15]	Iran	Retrospective cohort	116	84.5	43.54 ± 3.88	36		300	-
Hosseini et al. [14]	Iran	Retrospective cohort	138	84.8	45.35 ± 4.61	12		≥ 200	SASJ (24)

SD – Standard deviation; SASJ – Single anastomosis sleeve jejunal bypass

a - NIH (National Institutes of Health) quality assessment criteria for observational studies: it is based on a quality rating of G (good), F (fair), and P (poor), and 14 questions that can be answered with yes/no/not applicable/not reported/cannot determine. Y/N is the ratio of questions with positive answers (Y-yes) and negative answers (N–no)

*Common channel length was 250 cm in 18 patients, 300 cm in 88 patients, and 350 cm in 7 patients

**The length of the CL was determined according to the total bowel length (TBL). If the TBL was ≤ 6 m, the CL length was 250 cm. The CL length was 300 cm if the TBL was more than 6 m. 250 cm was performed 36% (116) of the time and 300 cm 64% of the time (206)

These studies were published between 2016 and 2022 and were conducted in Iran [14, 15, 22], United Arab Emirates (UAE) [17, 19], Egypt [5, 6, 20, 24–26], Poland [21], Mexico [23], India [8], Bahrain [9], and Turkey [7]. Two of the studies were multicentric, one was conducted in 7 countries (UAE, Kuwait, Egypt, Germany, Norway, Portugal, and Turkey) [18], and the other was in 2 countries (Egypt and UAE) [16].

A total of 1714 patients were studied, with sample sizes ranging from 19 to 551. The follow-up time ranged between 3 months and 36 months.

### Risk of bias of individual studies

The risk of bias in the observational studies is demonstrated in the Supplementary Table 2. Most studies failed to justify the sample size, and none ensured that the assessors were blinded to the exposure status. Only two studies had a loss of follow-up after baseline higher than 20%. For the other criteria, most studies showed a low risk of bias. Therefore, all studies were considered good or fair.

The results of the quality assessment of RCT are shown in Supplementary Table 3 [27]. Overall, this study was considered to have a low risk of bias since only two parameters were considered unclear.

### Comparison between SASI bypass and SG

#### Percentage of excess weight loss

**Fig. 2:**
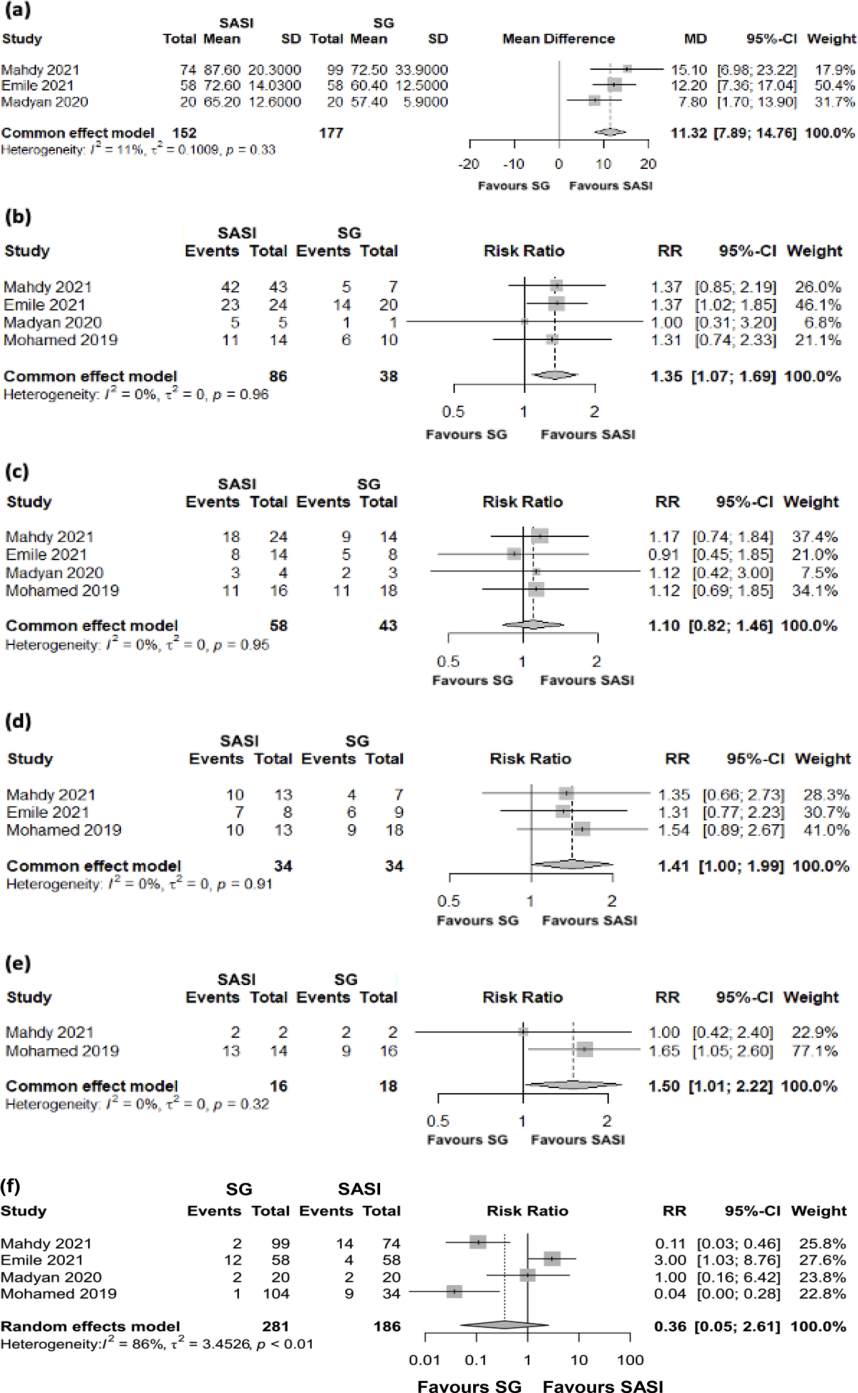
Forest plots of the comparation between SASI and SG; (**a**) %EWL, (**b**) T2DM, (**c**) HT, (**d**) DL, (**e**) OSA, and (**f**) complications

A total of 329 patients were analysed in three studies on %EWL after 12 months (Fig. 2a). SASI was found to be significantly better than SG in the meta-analytical %EWD MD (MD = 11.32; 95%CI[7.89;14.76]; *p* < 0.0001), with a moderate heterogeneity (*I*^*2*^ = 11%; *p* = 0.33). Heterogeneity ceased to be observed after removing the study by Madyan et al. (Supplementary Table 4).

#### Type 2 diabetes mellitus

A total of 124 patients were analysed in four studies that compared SASI versus SG on the partial or complete remission of T2DM after 12 months.

Compared with SG, SASI bypass was associated with 1.35 times more probability of improvement or resolution of T2DM, and this difference was significant (RR = 1.35; 95%CI[1.07; 1.69]; *p* = 0.011). No heterogeneity was detected (*I*^*2*^ = 0%; *p* = 0.96) (Fig. 2b).

#### Hypertension

Four studies compared SASI and SG on the improvement or resolution of HT in these two techniques (101 patients) after 12 months. The results presented in Fig. 2c show no significant difference (RR = 1.10; 95%CI[0.82;1.46]; *p* = 0.532) between the two approaches, and no heterogeneity was detected (*I*^*2*^ = 0%; *p* = 0.95).

#### Dyslipidemia

A total of 68 patients were analysed in two studies that compared partial or complete remission of DL after 12 months.

Compared to SG, a patient who underwent SASI bypass was associated with 1.41 times more probability of improvement or resolution of DL (RR = 1.41; 95%CI[1.00;1.99]; *p* = 0.048). No heterogeneity was detected (*I*^*2*^ = 0%; *p* = 0.91) (Fig. 2d).

#### Obstructive sleep apnea

A total of 34 patients were analysed in two studies that compared partial or complete remission of OSA after 12 months.

Compared to SG, a patient who underwent SASI bypass was associated with 1.5 times greater chance of improvement or resolution of OSA (RR = 1.50; 95%CI[1.01;2.22]; *p* = 0.042). No heterogeneity was detected (*I*^*2*^ = 0%; *p* = 0.32) (Fig. 2e).

#### Complications

A total of 467 patients were analysed in four studies after 12 months (Fig. 2f). No significant differences between techniques (RR = 2.81; 95%Cl[0.3;20.61]; *p* = 0.310) were observed, and high heterogeneity was detected (*I*^*2*^ = 86%; *p* < 0.01). Heterogeneity decreased (albeit remaining high – 67.1%) with the exclusion of the Emile et al. study (Supplementary Table 5).

### Comparison between SASI bypass and OAGB

In the comparison between SASI bypass and OAGB, it was not possible to establish a comparison regarding weight loss due to the lack of data.

#### Comorbidities

Two studies evaluated the improvement or remission of T2DM (110 patients), HT (81 patients), DL (50 patients), and OSA (33 patients) after 12 months. No significant differences between techniques were observed in T2DM (RR = 1.08; 95%CI[0.94;1.24]; *p* = 0.257), HT (RR = 0.87; 95%CI[0.69;1.11]; *p* = 0.266), DL (RR = 1.03; 95%CI[0.75;1.41]; *p* = 0.854), and OSA (RR = 1.14; 95%CI[0.85; 1.52]; *p* = 0.3789). No heterogeneity was detected in any of these.

#### Complications

**Fig. 3:**
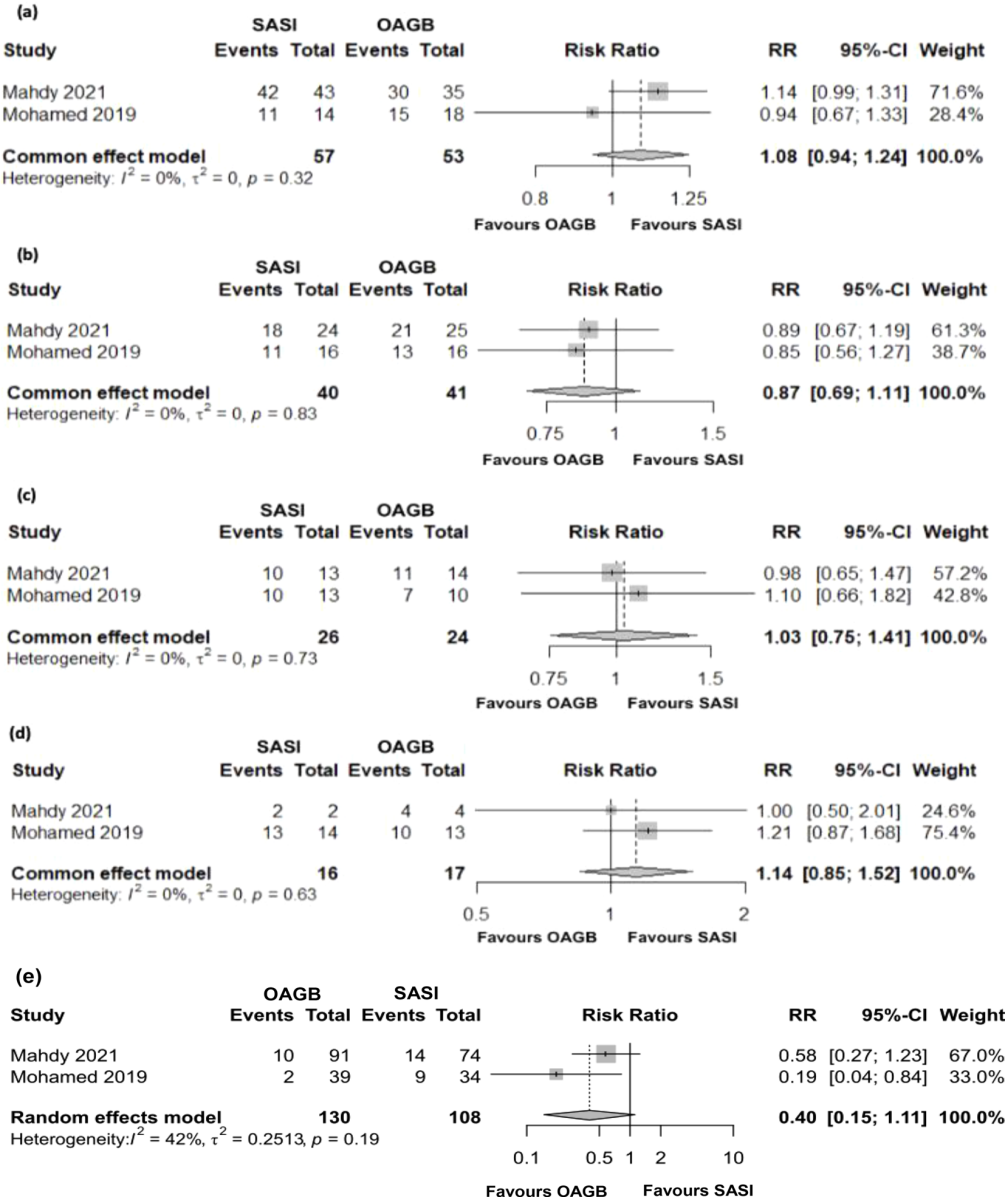
Forest plots of the comparation between SASI and OAGB; (**a**) T2DM, (**b**) HT, (**c**) DL, (**d**) OSA, and (**e**) complications

Four studies evaluated the complications in 283 patients after 12 months (Fig. 3e). No significant differences were observed between techniques (RR = 2.47; 95%CI[0.3;20.61]; *p* = 0.31), although moderated heterogeneity was observed (*I*^*2*^ = 42%; *p* = 0.19).

The effectiveness of SASI bypass, in terms of weight reduction and improvement in comorbidities, was associated with a favourable safety profile. In this review, complications were observed in 11.7% of patients, which is comparable to the rate reported in previous studies for SG [28]. Concerns regarding the need for reversal surgery were mainly due to excessive weight loss or hypoalbuminemia. However, in this review, the rates of excessive weight loss and hypoalbuminemia were 1.03% and 1.5%, respectively. Other complications reported were: 33 vomiting (~ 0,02%) [17, 18], 21 bleeding (~ 0,01%) [5, 6, 14–23, 25], 16 diarrhea (~ 0,01%) [18, 21], l0 leakage (~ 0,01%) [5, 6, 14, 15, 25], 8 stomal ulcer (~ 0,01%) [5, 6, 9, 18], 5 intestinal obstruction (< 0,01%) [5, 16–19], 4 pulmonary embolism (< 0,01%) [5, 15, 17, 18], 4 pneumonia (< 0,01%) [6, 16, 26], 4 dumping syndrome (< 0,01%) [6], 3 worsening symptoms of GERD (< 0,01%) [6, 20], 2 dysphagia (< 0,01%) [21, 23], 2 thrombosis (< 0,01%) [14], 2 wound infection (< 0,01%) [23], 2 peripheral neuropathy (< 0,01%) [19], 2 calcium deficiency (< 0,01%) [24], 2 internal hernia (< 0,01%) [6, 23], 2 calcular obstructive jaundice (< 0,01%) [18], 1 urinary tract infection (< 0,01%) [6], 1 ileal perforation (< 0,01%) [18], 1 illeus (< 0,01%) [26], 1 twisting of the biliopancreatic limb at the gastroileal anastomosis site (< 0,01%) [23], 1 biliary gastritis (< 0,01%) [23], 1 acute cholecystitis (< 0,01%) [23] and 1 trocar site hernia (< 0,01%) [22].

## Discussion

### Summary of evidence

The ideal bariatric surgery should be safe, technically simple, and successful in reducing weight and treating comorbidities [29]. The Santoro technique [30] was the foundation for SASI bypass, which had the goals of facilitating the procedure and try to lower the risk of complications, by reducing the number of anastomoses [5].

Most studies (14) used the %EWL to evaluate the impact of SASI bypass on the weight of participants [5, 6, 8, 14–23, 26]. The median %EWL at 12 months was 87.14%, which was nearly the same as in the initial report by Mahdy et al. (90%) [5]. A wide variation in the values was registered within the studies, varying between 63.9% and 94.33%. This deviation may be attributed to distinct patients’ characteristics, or a lack of technical standardization. Studies with a lower %EWL had a higher preoperative BMI. This is consistent with previous studies that showed that patients with a higher BMI had a lower %EWL after bariatric surgery. [31]. Comparing these results with a systematic review by Emile et al., which demonstrated that SG had a %EWL of 67.3% after 12 months [28], we can verify that SASI bypass has a better %EWL (87.14%), demonstrating a reasonable weight loss. These results are concordant with those in the comparison of SASI bypass with SG, indicating a clear superiority of SASI bypass. Due to a lack of data, we were unable to compare SASI bypass and OAGB in the meta-analysis. Two studies reported results after two years of follow-up, showing even better results (90.7% [15] and 96.7% [20]). Additionally, one study [15] had a three-year follow-up that had a mean %EWL of 80.6%.

In total, 713 patients were studied for the improvement or remission of T2DM after 12 months in 16 studies. The results ranged between 78.6% and 100%, with a median of 96.35% [5, 6, 8, 9, 14–23, 25, 26], which is higher than the results reported in previous studies for SG, RYGB, and OAGB [28, 32]. Although there was a wide range of results, only four studies had a rate lower than 90%. These results are concordant with those of our meta-analysis, which demonstrated that patients who underwent SASI bypass had better chances of improvement or resolution than those who underwent SG. Additionally, when compared with the OAGB, the SASI bypass had similar results.

Regarding HT, 15 studies evaluated the rate of complete remission and improvement, in 514 patients after 12 months [5, 8, 9, 14–23, 25, 26]. With a median of 75.8%, the findings were between 36.1% and 100%, with only five studies reporting an outcome under 75%. In our analysis, we determined that the SASI bypass had similar results compared to SG and OAGB.

In 11 studies, 340 patients with DL were evaluated after one year to calculate the proportion of full remission and improvement [5, 9, 14–19, 22, 23, 25]. The findings ranged from 65 to 100%, with a median of 87.5%, and only one study had a median under 75%.

According to our analysis, SASI bypass had higher chances of DL improvement or resolution than SG but had similar results compared to OAGB.

OSA was evaluated in eight studies that demonstrated an improvement or remission between 20% and 100%, with a median of 89.3% [14, 15, 17–20, 22]. Although the range of values was considerable, only three studies had a rate lower than 75%, and this difference could result from the distinct features between patients. The study that reported a value of 20% had a sample size of five patients with OSA, which can also explain the results. According to our meta-analysis, SASI bypass had better results than SG and similar results to OAGB.

Six studies evaluated GERD and reported a median improvement or remission rate of 84.1%, ranging from 75 to 100% [16, 18–20, 23, 26].

Owing to the limited sample size and lack of research, we were unable to establish a comparison for the meta-analysis of this outcome.

Two studies reported results regarding complete remission of non-alcoholic fatty liver disease (NAFLD) after 12 months. Kermansanavi et al. reported a rate of approximately 73% [22], and Hosseini et al. reported a rate of 90% [14]. Although these results are promising, further studies are needed to evaluate the efficiency of SASI bypass in NAFLD remission. In both studies, abdominal ultrasonography was performed to assess improvement in NAFLD.

Regarding the occurrence of biliary reflux, only one article reported a patient with evidence of severe biliary reflux on upper endoscopy, and he was managed by conversion to RYGB bypass [20]. No other evidence was found in any of the remaining articles. Nonetheless, biliary reflux should be addressed in future studies.

This review verified that technical variations in SASI bypass could induce different rates of weight loss, improvement in comorbidities and complications [28].

These variations included the length of the common limb, which varied between 200 and 350 cm; the size of the gastro-ileal anastomosis, which was 3–4 cm; and the gastro-ileal anastomosis proximal to the pylorus, which ranged between 3 and 6 cm.

Hosseini et al. demonstrated that a longer biliopancreatic limb was associated with greater improvement in HT, the cause of which is not clear. It may be related to alterations in the levels of ghrelin and GLP-1 hormones with respect to the length of the common limb, but further research is needed to better understand this mechanism [14].

On the other hand, a larger anastomosis size was associated with higher weight loss and a decrease in HT due to food diversion from the gastric pouch to the ileum, leading to an increasement in the malabsorptive effect [14].

Recently, a world consensus meeting was held in India, resulting in a statement suggesting standardization of numerous bariatric surgeries, including SASI bypass. Recommendations to standardise the SASI bypass method included a width of the residual sleeve of 3 cm, the gastric pouch between 150 and 250 cc, separating the gastroileal anastomosis from the pylorus by 2–6 cm, an anastomosis size of 3 cm, and a common limb length of 300 cm [33].

Throughout the studies, there were many advantages of the SASI bypass. The single anastomosis of SASI bypass has many advantages over a double anastomosis in the Santoro procedure (SG + TB), including being a simpler procedure, shorter operative time, and a lower risk of anastomosis complications and internal hernia.

Another advantage of this procedure is that it allows endoscopic inspection of the duodenum and biliary tract due to the preservation of natural endoscopic access.

Improvement in GERD symptoms after SASI bypass, compared to SG, can be explained by the addition of the anastomosis, which will reduce intragastric pressure and help drain gastric acidity, thus contributing to the improvement of symptoms [16, 17].

After some bariatric surgeries, patients have an excluded part of the stomach (i.e., RYGB, OAGB). This means that the detection of gastric lesions is challenging, and diagnosis and treatment may be delayed [34]. This is not a problem in SASI bypass, because it is a sleeve without an excluded stomach.

SASI bypass can be considered a technique between SG and SADI-S. Removing the anastomosis converts the SASI bypass to SG, and sectioning the duodenum converts the SASI bypass into a modified SADI-S. Therefore, it can be converted to SG in cases of malnutrition or excessive weight loss or modified to SADI-S if the bariatric and metabolic outcomes are considered unsatisfactory [35].

### Limitations

One of the main limitations of this review was the fact that only two studies [15, 20] had more than 12 months of follow-up, so there is a need for more studies with a longer follow-up period to evaluate mid-term and long-term outcomes. Additionally, a longer follow-up period is needed to guarantee that long-term complications such as malnutrition, hypoalbuminemia, and excessive weight loss will not develop.

Secondly, given that this technique is relatively recent, most studies had a limited number of participants. Therefore, most of the existing studies consist of small or medium-sized cohorts.

Owing to the small number of studies included in the SASI and OAGB meta-analyses, there are insufficient data to establish a significant comparison between these techniques.

A meta-analysis comparing SASI bypass and RYGB, the main bypass technique used, could not be established because only one of the selected studies compared these techniques.

There was not a common definition between studies in the definition of improvement/remission in comorbidities, which may cause some variation in results between studies and limits the conclusions presented related to comorbidities.

The high statistical heterogeneity obtained in some of the analyses, can be potentially explained by distinctive patient features, or lack of technical standardization.

Lastly, observational studies, which are more susceptible to bias and confounding variables, constituted nearly all the included studies. Consequently, determining a more effective technique can be more challenging. Therefore, more RCT are required.

## Conclusions

SASI bypass has a significant effect on weight loss, and remarkable metabolic control. Additionally, this new technique seems to be a safe bariatric surgical procedure. Nevertheless, there is a need for mid and long-term studies, comparing SASI to other better known and used choices (namely RYGB), in order to ascertain the true clinical advantage of SASI and determine the most robust factors for selecting patients for this procedure.

In conclusion, SASI bypass could be an important tool in the future, but there is a need for studies with longer follow-up periods (> 12 months) to confirm the efficiency and safety of this surgical technique.

### Electronic supplementary material

Below is the link to the electronic supplementary material.

Supplementary Material 1

## Data Availability

No datasets were generated or analysed during the current study.
